# Genetic and morphological evidence for introgression between three species of willows

**DOI:** 10.1186/s12862-015-0461-7

**Published:** 2015-09-16

**Authors:** Johan Fogelqvist, Alla V. Verkhozina, Alexander I. Katyshev, Pascal Pucholt, Christina Dixelius, Ann Christin Rönnberg-Wästljung, Martin Lascoux, Sofia Berlin

**Affiliations:** Swedish University of Agricultural Sciences, Department of Plant Biology, Uppsala BioCenter, Linnean Centre for Plant Biology, P.O. Box 7080, SE-75007 Uppsala, Sweden; Siberian Institute of Plant Physiology & Biochemistry, Irkutsk-33, P.O. Box 317, 664033 Irkutsk, Russia; Uppsala University, Department of Ecology and Genetics, Evolutionary Biology Centre, Science for Life Laboratory, Norbyvägen 18D, 752 36 Uppsala, Sweden

## Abstract

**Background:**

Hybridization and introgression are said to occur relatively frequently in plants, and in particular among different species of willows. However, data on the actual frequency of natural hybridization and introgression is rare. Here, we report the first fine-scale genetic analysis of a contact zone shared between the three basket willow species, *Salix dasyclados*, *S. schwerinii* and *S. viminalis* in the vicinity of the Lake Baikal in Southern Siberia. Individuals were sampled in fourteen populations and classified as pure species or hybrids based on a set of morphological characters. They were then genotyped at 384 nuclear SNP and four chloroplast SSR loci. The STRUCTURE and NewHybrids softwares were used to estimate the frequency and direction of hybridization using genotypic data at the nuclear SNP loci.

**Results:**

As many as 19 % of the genotyped individuals were classified as introgressed individuals and these were mainly encountered in the centre of the contact zone. All introgressed individuals were backcrosses to *S. viminalis* or *S. schwerinii* and no F1 or F2 hybrids were found. The rest of the genotyped individuals were classified as pure species and formed two clusters, one with *S. schwerinii* individuals and the other with *S. viminalis* and *S. dasyclados* individuals*.* The two clusters were significantly genetically differentiated, with *F*_*ST*_ = 0.333 (0.282–0.382, *p* < 0.001). In contrast, for the chloroplast haplotypes, no genetic differentiation was observed as they were completely shared between the species. Based on morphological classification only 5 % of the individuals were classified as introgressed individuals, which was much less than what was detected using genotypic data.

**Conclusions:**

We have discovered a new willow hybrid zone with relatively high frequency of introgressed individuals. The low frequency of F1 hybrids indicates that ongoing hybridization is limited, which could be because of the presence of reproductive barriers or simply because the conditions are not favorable for hybridization. We further conclude that in order to get a complete picture of the species composition of a hybrid zone it is necessary to use a combination of morphological characters and genetic data from both nuclear and chloroplast markers.

**Electronic supplementary material:**

The online version of this article (doi:10.1186/s12862-015-0461-7) contains supplementary material, which is available to authorized users.

## Background

Hybridization is a phenomenon that happens when mating between individuals from different species generates viable offspring and is a process that is estimated to occur in approximately 25 % of all plant species [1, 2]. Hybridization can sometimes lead to introgression if F1 hybrids backcross to one or both parental species and foreign genetic material is integrated into the genomes of either parent [3]. If hybrids or backcrossed individuals have increased fitness and develop reproductive isolation, hybridization and introgression can lead to diversification and speciation [3–5], which has been demonstrated in for example wild sunflowers and Louisiana irises [6]. In regions where previously geographically isolated species or populations meet, hybrid zones can form [7] if hybridization and introgression happens. Hybrid zones thus provide the opportunity to study evolutionary processes associated with interactions between species or differentiated populations. The genetic composition of hybrid zones depends to a large extent on the intensity of reproductive isolation and gene flow among interacting populations, which determines the frequency of F1 relative to introgressed F2 individuals [8–11].

In the past, hybrids were characterized solely based on morphology, however the use of morphological traits alone may not be sufficient to identify all hybrids or introgressed individuals [12], which was demonstrated by Hardig *et al*. [13] that failed to detect several hybrids between *Salix eriocephala* and *S. sericea* with only morphological characters. Therefore, in addition to morphological characteristics, molecular markers provide a good tool for assessing the degree of gene flow and genetic structure of hybrid zones. Due to the contrasting modes of inheritance of chloroplast and nuclear DNA they are expected to generate different patterns of genetic diversity within and among populations [14]. Thus a combination of nuclear and chloroplast markers can be used to disentangle the complex mode of gene flow in hybrid zones. For example, maternally inherited chloroplast markers allow the assessment of the maternal parent of the hybrids and the direction of hybrid matings. In addition, chloroplast DNA should due to its smaller effective population size, be more sensitive to genetic drift and therefore display higher levels of population differentiation than nuclear DNA [14].

Willows (*Salix*; Salicaceae) is a plant genus with tree and shrub species that are common in riparian habitats worldwide. The genus contains about 400 species [15], however due to an unusually complex taxonomy this number has varied greatly over time [16]. Willow species are notoriously difficult to classify as they are often morphologically similar and display large intraspecific diversity. In addition, hybridization might further complicate classification as hybrids can display unusual morphologies. The majority of willow species are diploid with a basis chromosome number of *n* = 19. However, ploidy levels can vary extensively both within and among species and range from diploids up to dodecaploids [17, 18] and possibly some of these polyploids are allopolyploids and the result of hybridization. The degree of hybridization has for centuries been a matter of great debate, and still is. In the late 1800’s and early 1900’s the general opinion was that hybridization was common and it was proposed that the great intraspecific morphological diversity was the result of hybridization [16]. Still, some taxonomists believed that the role of hybridization was greatly exaggerated and that many so called hybrids were not hybrids, but mere variants within species. In order to fully understand the role of hybridization for the evolution of willows, more studies are indeed needed. Nevertheless, there are several well-known cases of hybridization, for example between *S. fragilis* and *S. alba* in Europe [19], *S. sericea* and *S. eriocephala* in the U.S. [13] and between species in the subg. Vetrix e.g. *S. caprea, S. cinerea, S. atrocinerea* and *S. aurita* [20, 21].

**Fig. 1:**
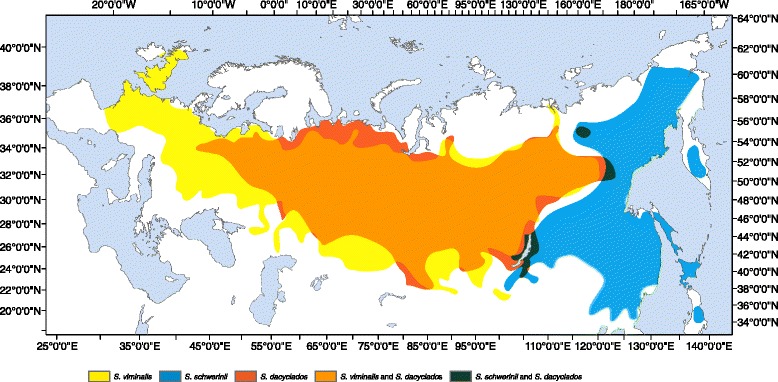
Distribution ranges of the three willows species. Modified from Skvortsov [16]. Orange represent *S. dasyclados*, blue represent *S. schwerinii* and yellow represent *S. viminalis*. Note the almost complete overlap between *S. dasyclados* and *S. viminalis* and the parallell distribution of *S. schwerinii*. The map was created using ArcGIS® software by Esri. ArcGIS® and ArcMap™ are the intellectual property of Esri and are used herein under license

*Salix viminalis* L., *S. dascyclados* Wimm. and *S. schwerinii* E. Wolf are closely related basket willows in the subg. Vetrix and are tall shrubs or trees found along streams and rivers and in other wet habitats [16]. *Salix viminalis* has a huge natural distribution [16], ranging from UK in the west to Siberia in the east (Fig. 1) and is the most well studied of the three species. It was in the past heavily used by humans for basketry and weaving but also for management of riverbanks and waterways [22]. Trading of cutting materials has contributed to the dispersal of the species, which is the most likely reason why it is present in Sweden, despite the fact that it is not naturally occurring in Scandinavia [23]. *Salix viminalis* and *S. dasyclados* are morphologically very similar and their distributions almost completely overlap (Fig. 1). According to Skvortsov [16], the two species have taxonomically been constantly confused and since hybrids between *S. viminalis* and other species of subg. Vetrix resemble *S. dasyclados*, *S. dasyclados* was also often regarded as a hybrid. However, Skvortsov [16] argued that no evidence exist that support a hybrid nature of *S. dasyclados. S. schwerinii* is found in East Asia and parallels *S. dasyclados* and *S. viminalis* in a north-south direction over a long distance across Siberia (Fig. 1). According to Skvortsov [16], *S. viminalis* and *S. schwerinii* meet in many geographical regions, however, they are rarely found growing together, and *S. schwerinii* is regarded as “vicarious” to *S. viminalis.* In a previous study, large genetic differentiation was found between the species as *F*_*ST*_ was estimated to 0.56 and coalescent simulations suggested a divergence time of around 600,000 years, however sampling was done outside of the contact zone [24].

Interspecific crosses between *S. viminalis × S. dasyclados* and *S. viminalis* × *S. schwerinii* have been produced experimentally and pure species as well as hybrids have been extensively used in pre-breeding programs aiming at producing high-yielding varieties for biomass production in Europe. For example, an experimental cross between *S. viminalis* × (*S. viminalis* × *S. schwerinii*) [25, 26] has been used in several QTL studies of different traits [27–33]. Interestingly in a crossing experiment between *S. viminalis* × *S. dasyclados* it was found that the *S. dasyclados* parent was hexaploid and the offspring tetraploid [34], indicating that some *S. dasyclados* individuals could be allopolyploids possessing genomes from several species. However, observations indicate that these three species are predominantly diploid.

In the present study we performed fine-scale genetic analysis of a contact zone with the three species *Salix dasyclados*, *S. schwerinii* and *S. viminalis* located in the vicinity of the Lake Baikal in Southern Siberia. The main objectives were to morphologically and genetically characterize individuals in the contact zone and to estimate the frequency and direction of hybridization using 384 nuclear SNP and four chloroplast SSR markers together with a set of morphological characters. Samples were collected in fourteen populations. We specifically asked the following questions: i) Is the contact zone a hybrid zone, that is, do we find hybrids or introgressed inidviduals? ii) If yes, what is the population structure of the hybrid zone? Do we primarily find intermediate F1 hybrids or introgressed individuals? iii) Is introgression uni- or bidirectional? iv) Can morphology be used to predict the structure of the hybrid zone? v) What are the characteristics of the reproductive barriers between the species?

## Results

### Morphological classification

A total of 375 individuals were collected from fourteen populations and based on morphological characterization nineteen individuals (5 %) were classified as hybrids.

**Fig. 2:**
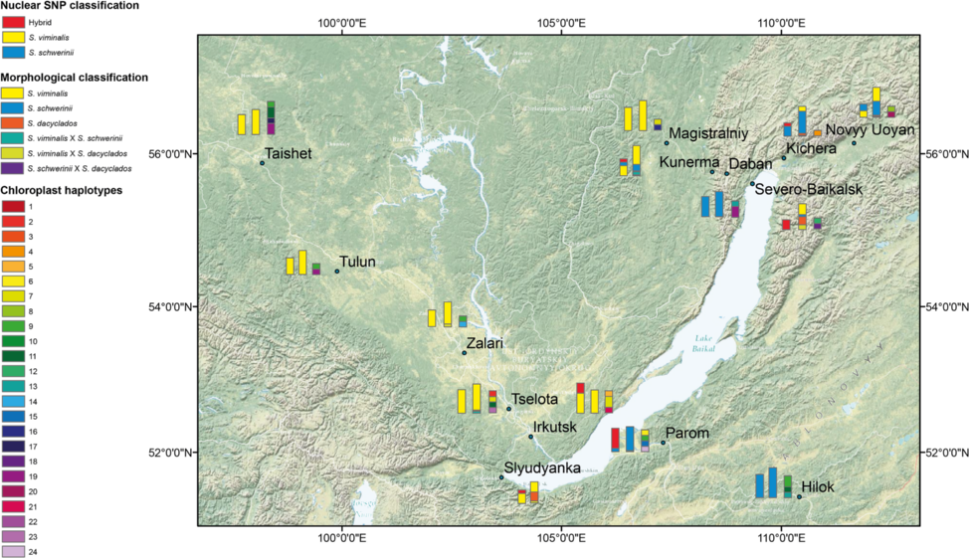
Population composition from analyses of nuclear SNP genotypes, chloroplast haplotypes and morphological characters. For each population there are three bars. The leftmost depict results from the cluster analysis with the STRUCTURE software on the nuclear SNP markers and show for every population the number of individuals assigned to cluster 1 (blue) and cluster 2 (yellow). The number of individuals with *Q*-values between 0.1 and 0.9 are shown in red. The middle bars demonstrate the results from the classification based in morphological characters and shows for every population the frequency of individuals classified as *S. viminalis* (yellow), *S. schwerinii* (blue), *S. dasyclados* (orange), *S. viminalis* × *S. schwerinii* hybrids (green), *S. viminalis* × *S. dasyclados* hybrids (lime) and *S. schwerinii* × *S. dasyclados* hybrids (purple). The rightmost bars demonstrate the occurrence of chloroplast haplotypes in every population. Note that Kunerma and Slyudyanka have only two bars, as no chloroplast haplotype data was available. The map was created using ArcGIS® software by Esri. ArcGIS® and ArcMap™ are the intellectual property of Esri and are used herein under license

Eleven were classified as *S. viminalis* × *S. dasyclados* hybrids, seven as *S. viminalis* × *S. schwerinii* hybrids and one as either a *S. viminalis* × *S. dasyclados* hybrid or a *S. schwerinii* × *S. dasyclados* hybrid (Additional file 1). Twenty-two individuals were classified as pure *S. dasyclados*, 123 as pure *S. schwerinii* and 209 as pure *S. viminalis* (for two individuals info was missing) (Additional file 1). Hybrids were encountered in eight different populations, two populations were composed of only *S. schwerinii* individuals and four populations were composed of only *S. viminalis* individuals (Additional file 1). The populations with hybrids were located in the central parts of the sampled area, pure *S. schwerinii* populations were located in the eastern parts and populations with only *S. viminalis* were located in the western parts (Fig. 2).

### Nuclear SNP variation and population structure

**Fig. 3:**
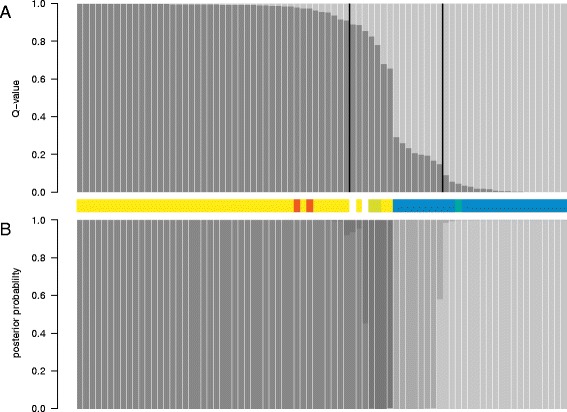
Identification and classification of hybrids. **a** STRUCTURE results showing the proportion of the genome of every individual originating from each of the two inferred clusters; medium-dark grey is cluster 1 (*S. viminalis*) and light grey is cluster 2 (*S. schwerinii*). The bars between the two vertical black lines show the introgressed individuals with *Q*-values between 0.1 and 0.9. **b** NewHybrids classification for every individual with posterior probabilities for a given class; medium-dark grey is the *S. viminalis* class, light-grey is the *S. schwerinii* class, dark grey is the class with backcrosses to *S. viminalis* and medium-light grey is the class with backcrosses to *S. schwerinii*. The morphological classification of each individual sample is shown in the middle, colour coded as in Fig. 2 (*S. viminalis*: yellow, *S. schwerinii*: blue, *S. dacyclados*: orange, *S. viminalis* x *S. schwerinii*: green, *S. viminalis x S. dacyclados*: lime, Infomation missing: white)

Seventy-nine individuals were successfully genotyped at 323 of the 384 nuclear SNP loci, of which 206 were polymorphic in at least one of the individuals. Both pure species and hybrids were present among these 79 individuals (two were classified as *S. dasyclados*, 45 as *S. viminalis*, 27 as *S. schwerinii*, two as *S. viminalis* × *S. dasyclados* hybrids and one as a *S. schwerinii* × *S. dasyclados* hybrid (for two individuals info was missing), thus they form a representative subset of all collected samples. Two SNPs in *S. schwerinii* and three in *S. viminalis* deviated significantly from Hardy-Weinberg equilibrium (*p* ≤ 0.01) (Additional file 2). One hundred and forty-six SNPs were polymorphic in both species, 40 were polymorphic only in *S. viminalis* and 20 were polymorphic only in *S. schwerinii*. No fixed SNPs were found. *F*_*ST*_ was estimated to 0.057 (AMOVA, 95 % CI: 0.033–0.075, *p* < 0.001) in *S. viminalis*, 0.101 (AMOVA, 95 % CI: 0.079–0.123, *p* < 0.001) in *S. schwerinii* and 0.333 between the species (0.282–0.382, *p* < 0.001). When all samples were included in clustering analysis with the STRUCTURE software [35–38], the number of clusters (*K*) was estimated to 2, both when the original method from Pritchard et al. [35] was used and with the ΔK statistics given in Evanno et al. [39] (Additional file 3). Sixty-four individuals had *Q*-values ≥ 0.9 and 44 of these were assigned to cluster 1 and twenty were assigned to cluster 2 (Fig. 3a). Based on the morphological classification, all but two individuals that originated from cluster 1 were *S. viminalis*. The two other individuals were *S. dasyclados*, meaning that genetically *S. dasyclados* and *S. viminalis* are indistinguishable. In cluster 2, nineteen out of the twenty individuals were morphologically *S. schwerinii* and one was a *S. viminalis* × *S. schwerinii* hybrid. Individuals of *S. viminalis* origin were found in populations in the western parts of the sampled area and individuals with *S. schwerinii* origin were found in populations in the eastern parts (Fig. 2).

### Hybrid identification

Fifteen of the genotyped individuals (19 %) had *Q*-values between 0.1 and 0.9 and were identified as hybrids (Table 1; Fig. 3a; Additional file 4). These individuals derive from six populations located in the centre of the sampling area (Fig. 2). Morphologically, only two were classified as hybrids (*S. viminalis* × *S. dasyclados*), while eight were classified as *S. schwerinii* and three as *S. viminalis* (for two others, morphological classification was missing) (Table 1). As no *Q*-value was close to 0.5 (Table 1), this was interpreted as no F1 hybrid was present among the individuals. Instead, their *Q*-values suggest that they were backcrosses both to *S. viminalis* and *S. schwerinii*. Thirteen of the fifteen introgressed individuals had posterior probabilities > 0.9 (Table 1; Fig. 3b), of which seven were classified as *S. schwerinii* backcrosses, four as *S. viminalis* backcrosses and two as pure *S. viminalis*, while none was classified as F1 or F2 hybrids (cross between two F1s).

**Table 1:** Classification based on *Q*-values in STRUCTURE, posterior probabilities in NewHybrids and morphological characters

Sample	Proportion *S. viminalis* (*Q*-value)	Proportion *S. schwerinii* (*Q*-value)	Classification with STRUCTURE	Classification with NewHybrids (Posterior probability)	Morphological classification
I2	0.889	0.111	*S. viminalis* backcross	*S. viminalis* (0.934)	*No info*
I6	0.856	0.144	*S. viminalis* backcross	*S. viminalis* (0.449), *S. viminalis* backcross (0.551)	*No info*
I16	0.887	0.113	*S. viminalis* backcross	*S. viminalis* (0.955)	*S. viminalis*
Ki28	0.293	0.707	*S. schwerinii* backcross	*S. schwerinii* backcross (1)	*S. schwerinii*
Ku20	0.152	0.848	*S. schwerinii* backcross	*S. schwerinii* (0.569) *S. schwerinii* backcross (0.431)	*S. schwerinii*
P1	0.195	0.805	*S. schwerinii* backcross	*S. schwerinii* backcross (1)	*S. schwerinii*
P4	0.169	0.831	*S. schwerinii* backcross	*S. schwerinii* backcross (1)	*S. schwerinii*
P8	0.208	0.792	*S. schwerinii* backcross	*S. schwerinii* backcross (1)	*S. schwerinii*
P12	0.201	0.799	*S. schwerinii* backcross	*S. schwerinii* backcross (1)	*S. schwerinii*
P16	0.237	0.763	*S. schwerinii* backcross	*S. schwerinii* backcross (1)	*S. schwerinii*
P20	0.264	0.736	*S. schwerinii* backcross	*S. schwerinii* backcross (1)	*S. schwerinii*
S14	0.829	0.171	*S. viminalis* backcross	*S. viminalis* backcross (1)	*S. viminalis × S. dasyclados*
SB4	0.656	0.344	*S. viminalis* backcross	*S. viminalis* backcross (1)	*S. viminalis*
SB6	0.682	0.318	*S. viminalis* backcross	*S. viminalis* backcross (1)	*S. viminalis*
SB17	0.780	0.220	*S. viminalis* backcross	*S. viminalis* backcross (1)	*S. viminalis × S. dasyclados*

### Chloroplast SSR variation

Seventy individuals originating from Europe and the Baikal region were successfully genotyped with the four chloroplast SSR markers. As expected, one allele per locus and individual was found for each of the markers. A total of seventeen alleles were detected and *ccmp2* was the most variable with six alleles and *ccmp4* the least variable with two alleles, *ccmp5* had four and *ccmp6* had five alleles. When combined, these alleles formed 24 different haplotypes. When only including the individuals from the Baikal region (*N* = 50) the *PhiPT* (an analog to *F*_*ST*_ , i.e genetic differentiation among populations) was estimated to -0.023 (*p* = 0.647) in *S. viminalis*, to 0.107 (*p* = 0.100) in *S. schwerinii* and to -0.006 (*p* = 0. 371) between the species. Thus, the chloroplast haplotypes did not display any significant genetic differentiation, either within or between the species (Fig. 2; Additional file 5). No sequence variation was found in the two sequenced chloroplast regions (*trnL* and *rbcL*).

## Discussion

The three closely related basket willow species *Salix dasyclados*, *Salix schwerinii* and *Salix viminalis* are phenotypically similar and several morphological characteristics are needed to distinguish them from each other. Given that the three species recently diverged, it is possible that they would hybridize where they come into contact in nature, unless some strong reproductive barriers have evolved. To study this, individuals from all three species were sampled in fourteen populations in a region in Southern Siberia where the distributions of the species meet and overlap. 81 % of the genotyped individuals were assigned to one of two clusters; one composed of *S. schwerinii* and one of *S. viminalis* and *S. dasyclados* individuals*.* Relatively high level of genetic differentiation was observed between the clusters (*F*_*ST*_ of 0.333), suggesting that they are largely reproductively isolated. However, 19 % of the genotyped individuals were identified as hybrids, which shows that the species boundaries are partly permeable. Both the STRUCTURE and the NewHybrid analyses showed that none of these individuals were intermediate F1 hybrids but backcrosses to either *S. schwerinii* or *S. viminalis*. This observation demonstrates that introgression is bidirectional in this willow hybrid zone. Furthermore, the absence of F1 hybrids is striking and is best explained by the presence of pre- and/or postmating reproductive barriers that form reproductive isolation between the species [40]. Currently, we can only speculate about the nature of these barriers in this system and the relative contribution of different barriers. Putative premating barriers are differences in flower phenologies or pollinator specificity [41] and as willows generally are both wind and insect pollinated [42, 43], both are likely to be involved. We do not know whether or not *S. schwerinii* and *S. viminalis* display asynchronous flowering in the region of sampling. However, at our field site outside of Uppsala in Sweden (59.80° N, 17.67° E, 25 m AOD), *S. schwerinii* plants flower considerably earlier than *S. viminalis* plants, bearing in mind that those *S. viminalis* plants originate from Central Europe and not from the region near Lake Baikal. Possibly, postmating barriers are operating together with premating barriers to produce strong reproductive isolation, as premating barriers alone are often permeable and leaky [41]. However as artificial crosses between the species are routinely produced in our laboratory and F1 hybrids are fertile and also used to backcross to either parents, strong postmating barriers are unlikely to be present. The age of the hybrid zone may also affect the occurrence of F1 hybrids as in a very young zone, not enough time may have elapsed for intermediate hybrids to reach larger numbers. However as we found a substantial number of backcrossed individuals, the hybrid zone could not be very young. Perhaps instead, the hybrid zone is relatively old and the intermediate hybrids have been lost as they are no longer being produced or produced at very low frequencies. It could also be, that environmental conditions and the relative proportion of species [44] were conducive to hybridization during the formation of the hybrid zone but are no longer so.

An interesting observation was that chloroplast haplotypes were shared between the species and no genetic differentiation was found, even when including samples from Europe. This could indeed be the result of homoplasy, however as extensive haplotype sharing has been reported in other willow [13, 45, 46] and tree species, e.g. *Betula* spp. [47] and *Quercus* spp. [48] using entirely different markers, it is unlikely that homoplasy alone could create the observed pattern. Instead, haplotype sharing is likely the result of past hybridization and/or introgression. The observation that none of the SNPs were fixed between the species furthermore supports the scenario that introgression happens where the two species meet. While this may well be the case, our data also suggest that to some degree the shared haplotypes and polymorphisms are ancestral polymorphism. Shared ancestral polymorphism between species is expected from incomplete lineage sorting, which is particularly likely in species such as the willows that have large effective population sizes as it under those circumstances take very long time for alleles to go to fixation [49]. In a previous study, using sequences from *S. viminalis* and *S. schwerinii* individuals from Europe and Siberia, respectively, i.e. long way from the hybrid zone, divergence was estimated at 600,000 ya [24]. The effective population size in our previous study was estimated to 40,000 in both species and the generation time assumed to be 10 years. Thus, the divergence time would be roughly 1.5 Ne and would be indeed insufficient to observe reciprocal monophyly. This shows that even if gene flow is not currently taking place, we expect to observe shared polymorphism due to historical gene flow and/or incomplete lineage sorting.

The chloroplast and nuclear DNA gave contrasting results as there was no significant genetic differentiation with the chloroplast haplotypes, while with the nuclear genotypic data there was relatively high level of genetic differentiation. This pattern is a likely consequence of the differences in intraspecific gene flow that the nuclear and the chloroplast DNA experience [44]. Since nuclear DNA is transmitted by both pollen and seeds and as chloroplast DNA is transmitted only by seeds, intraspecific gene flow will be higher for nuclear DNA compared to chloroplast DNA. Introgressed nuclear alleles will therefore be diluted and maintained at low frequencies, however introgressed chloroplast haplotypes will not be diluted to the same degree and can therefore persist in the population. This effect should be particularly significant in species where pollen can travel long distances by wind and where seeds are less prone to long distance dispersal. Willow seeds are also primarily wind dispersed, [50], however compared to pollen, seeds are expected to travel less far.

Compared to the 19 % of the genotyped individuals that were assessed to be of hybrid origin, only 5 % were classified as hybrids using traditional morphological characteristics. Most noteworthy was that eight individuals that were classified as pure *S. schwerinii* were in fact introgressed individuals. This demonstrates the difficulties and uncertainties in detecting hybrids solely based on morphology, which could be particularly difficult at low levels of introgression. Another surprising finding was that individuals classified as *S. dasyclados* clustered with individuals classified as *S. viminalis*. As we were unable to distinguish the species from each other with this large set of markers, one conclusion could be that the sampled individuals should be regarded as one and the same species. Still, they display enough morphological differences to be classified as different species by trained botanists. In order to solve this, more samples need to be analysed, both genetically and morphologically.

## Conclusions

We have discovered a new hybrid zone between three willow species with rather high frequency of introgressed individuals, which were identified both with morphological characters and genotypic data. We found extensive sharing of chloroplast haplotypes between the species and a large number of shared nuclear polymorphisms, a pattern that is expected from introgression but also from ancestral polymorphisms that remain in the populations due to incomplete lineage sorting. We also demonstrated rather high levels of reproductive isolation between *S. viminalis* and *S. schwerinii*. This was further supported by the lack of intermediate F1 hybrids, which could be a consequence of some of reproductive barrier(s). In addition we found that introgression was bidirectional so that backcrosses happen with both *S. schwerinii* and *S. viminalis*. Another surprising observation was the genetic similarities between *S. dasyclados* and *S. viminalis*, which leads us to doubt the taxonomic status of *S. dasyclados*.

## Methods

### Sampling and morphological classification

Shoots and leaves from *S. dasyclados*, *S. schwerinii* and *S. viminalis* and putative hybrids were collected from plants in the field in July 2012. Samples were collected in fourteen different populations (Table 2 and Fig. 2) in the vicinity of the Lake Baikal in Southern Siberia, Russia. In each population, between 25 and 30 individuals were collected (Additional file 1). If all species were present in one population, the number of individuals collected per species approximately corresponded to the relative abundance of that species to the other. In the laboratory, a herbarium collection was established and each individual was carefully examined and classified as pure species or hybrids. The species and hybrids were morphologically discriminated using a set of characters that are listed in Additional file 6. Leaf tissue samples for each individual was prepared, dried in silica gel and shipped to Uppsala, Sweden for genetic analyses.

**Table 2:** Description of the fourteen populations in the Lake Baikal region

Population	Coordinate (Location by Google Map)	No of ind. sampled	No of ind. genotyped for nuclear SNPs and chloroplast SSRs (in brackets)	Ancestry based on nuclear SNPs (no. of individuals)
Taishet (Ta)	55.877237, 98.187853	25	6 (6)	*S. viminalis* (6)
Tulun (T)	54.470038, 99.89245	24	5 (2)	*S. viminalis* (5)
Zalari (Z)	53.36958, 102.784003	25	5 (4)	*S. viminalis* (5)
Slyudyanka (S)	51.643164, 103.64217	21	4 (1)	*S. viminalis* (3), mixed (1)
Tselota (C)	52.605015, 103.806786	29	7 (5)	*S. viminalis* (6)
Irkutsk (I)	52.218427, 104.306664	25	9 (5)	*S. viminalis* (6), mixed (3)
Parom (P)	52.134226, 107.3165	25	7 (4)	*S. schwerinii* (1), mixed (6)
Magistralniy (M)	56.132829, 107.392632	30	7 (6)	*S. viminalis* (7)
Kunerma (Ku)	55.766483, 108.432821	30	5 (1)	*S. schwerinii* (1), *S. viminalis* (3), mixed (1)
Daban (D)	55.743395, 108.755881	25	6 (5)	*S. schwerinii* (6)
Severo-Baikalsk (SB)	55.616486, 109.341853	26	3 (2)	Mixed (3)
Kichera (Ki)	55.942567, 110.060851	30	4 (2)	*S. schwerinii* (3), mixed (1)
Hilok (H)	51.369262, 110.407227	30	7 (3)	*S. schwerinii* (7)
Novyy Uoyan (N)	56.131011, 111.651967	30	4 (3)	*S. schwerinii* (2), *S. viminalis* (2)

### DNA extractions and genotyping

Genomic DNA from dried leaf tissue samples was isolated using the DNeasy Mini Plant Kit (Qiagen, Crawley, UK). Genotypes were determined at 384 previously developed single nucleotide polymorphism (SNP) loci [25] with the Illumina GoldenGate Assay at the SNP&SEQ Technology Platform, Science for Life Laboratory at Uppsala University, Sweden. The SNP markers were identified in *S. viminalis* and *S. schwerinii* individuals and have random genomic locations [25]. Alleles were scored at four chloroplast SSR loci; ccmp2, ccmp4, ccmp5 and ccmp6 [51]. The chloroplast SSR markers were amplified by PCR, multiplexed and separated on an ABI3730XL instrument at the Uppsala Genome Center, Science for Life Laboratory at Uppsala University, Sweden. The PCRs were performed in 10 μl reactions with 10 ng of DNA, 1xPCR buffer (Qiagen, Stockholm, Sweden), 0.75 mM MgCl_2_ (Qiagen, Crawley, UK), 0.9 mM of a mix of dNTPs (Applied Biosystems, Stockholm, Sweden), 0.1 μM of each primer (Life Technologies, Stockholm, Sweden) and 0.25 U Taq HotStar Plus (Qiagen, Stockholm, Sweden). The PCR program included 95 °C for 5 min, then 30 cycles at 95 °C for 30 s, T_m_ for 30 s, 72 °C for 30 s, then 8 °C on hold. T_m_ was 58 °C for ccmp2, ccmp4 and ccmp6 and 56 °C for ccmp5. The forward primers were fluorescently labeled at the 5´end; ccmp2 with 6-FAM, ccmp4 with NED, ccmp5 with VIC and ccmp6 with PET. Allele sizes were determined with the PeakScanner ^TM^ Software v1.0 (Life Technologies, Stockholm, Sweden). In addition to individuals sampled in the fourteen populations in Southern Siberia another twenty samples, morphologically classified as *S. viminalis* were scored with the chloroplast SSRs; four originated from western Russia and sixteen originated from Europe. Two chloroplast regions were sequenced and analysed for sequence variation; an intron in the tRNA-Leu gene (*trnL*) amplified with primers c and d [52] and a fragment in the *rbcL* gene (Rubisco) amplified with primers carbcLF and carbcLR [53]. The PCRs were performed in 30 μl reactions with approximately 10 ng of DNA, 1xPCR buffer (Qiagen Crawley, UK), 2 mM MgCl_2_ (Qiagen Crawley, UK), 0.8 mM of a mix of dNTPs (Applied Biosystems Thermo Fisher Scientific, Stockholm, Sweden), 0.2 μM of each primer (Life Technologies Thermo Fisher Scientific, Stockholm, Sweden) and 1.5 U Amplitaq GOLD (Applied Biosystems, Stockholm, Sweden). The PCR program included 94 °C for 5 min, five cycles of 94 °C for 30 s, 65-60 °C for 30 s, (- 1 °C every cycle), then 25 cycles at 94 °C for 30 s, 60 °C for 30 s and 72 °C for 90 s, then 72 °C for 10 min and finally 10 °C on hold. Amplification was determined by agarose gel electrophoresis. PCR products were cleaned and sequenced at Macrogen Inc. (Macrogen, Seoul, South Korea) on both strands using the forward and reverse PCR primers as sequencing primers. The chromatograms were visually inspected and edited using Lasergene SeqMan vs 9.1.0 (DNASTAR).

### Genetic analyses

The R package [54] adegenet v1.4-2 [55, 56] was used to calculate observed and expected heterozygosity and departure from Hardy-Weinberg equilibrium for each of the nuclear SNP markers. Genetic divergence was estimated as *F*_*ST*_ both within and between *S. schwerinii* and *S. viminalis* populations using Arlequin v. 3.5 [57]. Shared and fixed number of SNP loci between and within *S. schwerinii* and *S. viminalis* were estimated. For these analyses, individuals were grouped based on morphological characteristics and the hybrids and *S. dasyclados* were not included.

Genetic differentiation was investigated using Bayesian cluster analyses with the STRUCTURE software v 2.3.2.1 [35–38]. An admixture model was employed where correlated allele frequencies were assumed, and the *K*-value (i.e. the number of clusters) was set from one to eight, a value within this range being expected to be the most likely result. The length of the burn-in period was set to 10,000 and the Monte Carlo Markov Chain (MCMC) model after burn-in was run for an additional 100,000 iterations. For each *K*, 40 replicates were run and runs with an outlier value of *lnPD* were removed (*χ*^*2*^-test, α = 0.05, as implemented in R-package outliers). The optimal value of *K* was determined by examination of the *L*(*K*) and Evanno’s *∆K* statistics [39] using the R package ‘CorrSieve’ [58].

For each of the four chloroplast SSR markers, allele numbers and size ranges were assessed. Alleles were combined in haplotypes for every individual. *PhiPT* within and between *S. schwerinii* and *S. viminalis* was assessed using GenAIEx v 6.5 [59, 60]. Haplotypes in each population were plotted in Fig. 2. All maps were created using ArcGIS® software by Esri. ArcGIS® and ArcMap™ are the intellectual property of Esri and are used herein under license. Copyright © Esri. All rights reserved. For more information about Esri® software, please visit www.esri.com.

### Identification and classification of hybrids

Hybrids and backcrosses were identified using the nuclear genotypic data and the softwares STRUCTURE [35–38] and NewHybrids [61, 62]. The *Q*-value from STRUCTURE, which shows the proportion of an individual’s genome that originates from each of the *K* clusters, was used as hybrid index (similar to what was done in Devitt et al. [63]). Thus, for every *K*, individuals with *Q*-values between 0.1 and 0.9 were considered to be hybrids or backcrosses, while individuals with *Q*-values ≥ 0.9 were considered to be pure species. NewHybrids was used to classify individuals as pure species, F1s, F2s or backcrosses. NewHybrids computes the posterior probabilities that an individual belongs to these different classes. For NewHybrids, we used the default genotype categories for first- and second-generations of crossing and ran 100,000 sweeps of five chains started from overdispersed starting values after a burn-in period of 50,000 sweeps following the software author’s recommendation. Jeffrey-type priors were used for the mixing proportions and allele frequencies. To check for convergence, we visually inspected P(z) values from the different runs which were then averaged across the 5 runs.

### Availability of supporting data

The data set supporting the results of this article is archived in LabArchives (doi: 10.6070/H48P5XJ2).

## Additional files

Additional file 1:
**Collected samples and classification.** (XLSX 14 kb) Additional file 2:
**H**_**O**_ **and H**_**E**_ **and test of Hardy-Weinberg equilibrium for every marker for** ***S. schwerinii*** **and** ***S. viminalis*** **separately.** (XLSX 52 kb) Additional file 3:
**lnPD and deltaK from the Bayesian cluster analyses with the STRUCTURE software.** (PDF 672 kb) Additional file 4:
***Q*** **-values with K = 2 (STRUCTURE) and posterior probabilities (NewHybrids).** (XLSX 15 kb) Additional file 5:
**Chloroplast DNA haplotype distribution across Europe and Russia.** (PNG 236 kb) Additional file 6:
**Morphological characters used to classify species as** ***S. dasyclados***, ***S. schwerinii*** **or** ***S. viminalis***. (XLS 25 kb)
