# Supplementary figures and images for: Genetic and morphological evidence for introgression between three species of willows
Source: BMC Evol Biol. 2015 Sep 16;15:193. doi: 10.1186/s12862-015-0461-7 (PMC4574262; doi:10.1186/s12862-015-0461-7)

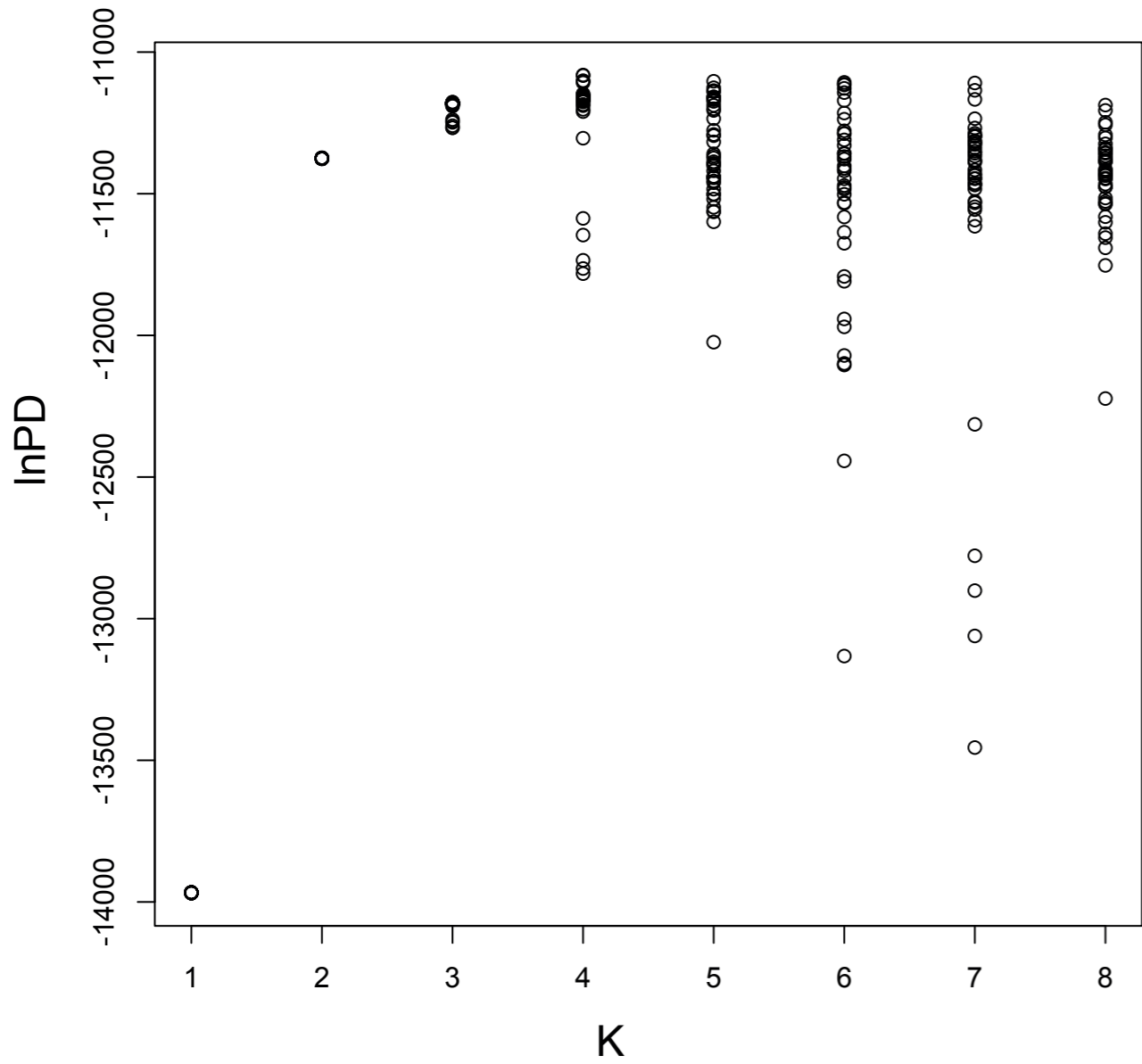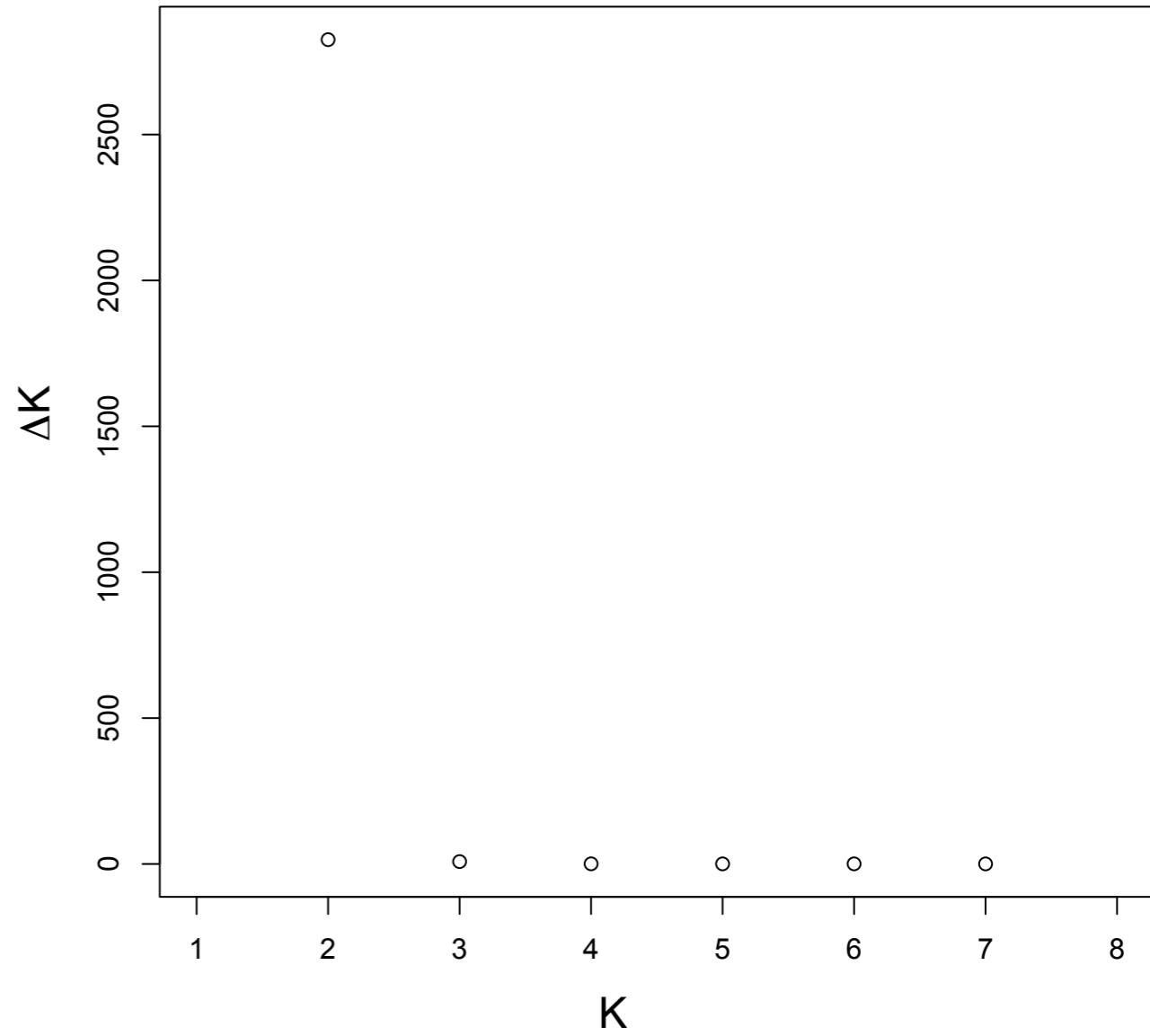

Supplement: Additional file 3: — lnPD and deltaK from the Bayesian cluster analyses with the STRUCTURE software. (PDF 672 kb) [file 12862_2015_461_MOESM3_ESM.pdf]

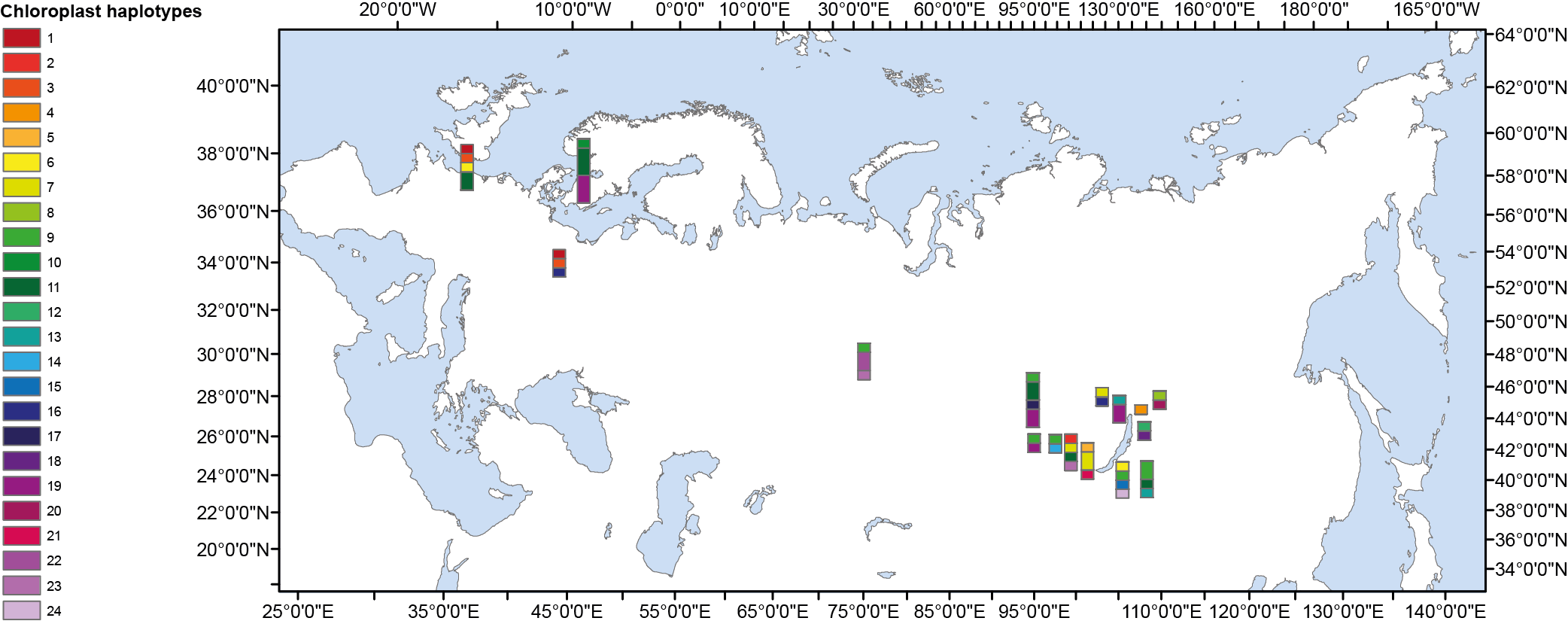

Supplement: Additional file 5: — Chloroplast DNA haplotype distribution across Europe and Russia. (PNG 236 kb) [file 12862_2015_461_MOESM5_ESM.png]
